# Construction of a digital literacy education support model and development and validation of a scale for physical education majors in China

**DOI:** 10.3389/fpsyg.2026.1901994

**Published:** 2026-10-09

**Authors:** Dongbin Lai, Zhi Li, Shufeng Zhang, Pengfei Wen

**Affiliations:** 1 School of Physical Education, Guangzhou Sport University, Guangzhou, China; 2 Division of Sports Science and Physical Education, Tsinghua University, Beijing, China

**Keywords:** digital literacy, educational support, grounded theory, physical education majors, scale development

## Abstract

**Introduction:**

Physical education majors have a dual identity as current learners and future educators. Their digital literacy is not only related to their adaptation to digital learning environments but also directly influences the development of their future competence in physical education teaching, training organization, and management practice. Therefore, it is necessary to systematically identify and strengthen educational support for their digital literacy development. However, existing studies have mainly focused on digital literacy levels or single influencing factors, with insufficient attention paid to the educational support structure underlying the formation of digital literacy. In particular, standardized measurement tools tailored to the context of physical education remain limited. This study aimed to construct a Digital Literacy Education Support model for physical education majors in China and to develop and validate a corresponding scale.

**Methods:**

Semi-structured interviews were conducted with 22 physical education majors and 12 university teachers and counselors. The interview data were analyzed using grounded theory through three-stage coding, which identified five main categories: personal traits, symbiotic environment, school environment, social environment, and policy and institutional system. Based on these categories, an educational support model was constructed. Subsequently, an initial item pool was developed through literature analysis and expert review, and item analysis, exploratory factor analysis, confirmatory factor analysis, and reliability and validity testing were conducted using pilot and formal survey data.

**Results:**

The results showed that exploratory factor analysis supported a five-factor structure, explaining 58.119% of the total variance. Confirmatory factor analysis indicated good model fit (*χ*^2^/df = 2.725, RMSEA = 0.037, CFI = 0.968, TLI = 0.965, IFI = 0.968). Cronbach’s alpha coefficients, split-half reliability, and test–retest reliability for all dimensions met recommended psychometric standards, and the scale demonstrated good convergent and discriminant validity.

**Discussion:**

The findings suggest that the development of digital literacy among physical education majors is shaped by the joint effects of multilevel support involving individual, relational, school, social, and institutional conditions. The developed 30-item scale shows good reliability and validity and can provide a theoretical basis and practical tool for universities to diagnose and improve educational support for digital literacy among physical education majors.

## 1 Introduction

With the rapid development of the digital era, digital technologies represented by artificial intelligence, big data, and virtual reality are profoundly reshaping modes of social production and knowledge dissemination (Mukul and Büyüközkan, 2023). Digital literacy (DL) has become an essential competence for individuals to adapt to the digital age and has been recognized by international organizations such as the Organisation for Economic Co-operation and Development (OECD) and the United Nations Educational, Scientific and Cultural Organization (UNESCO) as one of the core competencies for citizens in the 21st century (OECD, 2023; UNESCO Institute for Statistics, 2018). DL is not limited to basic operational skills in using digital tools; rather, it emphasizes a comprehensive capacity that includes information access and evaluation, problem solving and critical thinking, as well as creative expression and practical application through digital technologies. Meanwhile, educational digitalization strategies have clearly highlighted the need to improve the DL and skills of teachers and students. As key participants in and beneficiaries of this strategy, university students’ level of DL directly influences the effectiveness of educational digitalization (Timotheou et al., 2023; Li and Du, 2026).

The development of university students’ DL varies across disciplinary contexts. Physical education majors have a dual identity as current learners and future educators. Their DL is not only related to their ability to adapt successfully to digital learning environments, but also directly affects their future competence in diverse professional tasks such as physical education teaching, training organization, and sport management. These tasks increasingly involve the use of technologies such as sport data analysis, wearable devices, and online teaching platforms, thereby placing higher demands on physical educators’ abilities in instructional design, process monitoring, and data processing. For physical education majors who are about to enter diverse professional positions, DL is no longer an additional competence, but a foundational capacity for adapting to digital learning and meeting the demands of future professional practice (Ferrari, 2013; Salimzyanova et al., 2021). However, the overall DL of physical education majors remains relatively weak and is insufficient to meet the dual demands of current learning adaptation and future professional practice (He, 2026). Therefore, how to systematically promote the development of DL among physical education majors has become an urgent issue to be addressed.

To answer this question, it is first necessary to clarify which factors can support the development of DL among physical education majors. Existing studies have examined DL and its influencing factors; however, several limitations remain. In terms of research participants, most studies have focused on groups such as pre-service teachers or in-service teachers (Li et al., 2024; Wang and Li, 2023; Xie et al., 2025; Martínez-Rico et al., 2022), with insufficient attention paid to the specific context of physical education. In terms of theoretical perspective, existing research has mainly examined DL from the perspective of single factors (Li et al., 2024; Fang and Zhang, 2024; Fu and Zhou, 2025; Wohlfart et al., 2024; Wallace et al., 2023; Alano et al., 2025), lacking a systematic integration of the multidimensional elements that promote DL development and thus failing to reveal its overall mechanism. In terms of measurement tools, existing studies have primarily focused on assessing DL competence (Xie et al., 2025; Xu, 2025; Avinç and Doğan, 2024; Anisimova, 2020), while paying insufficient attention to the structure of educational support in the formation of DL. In particular, there remains a lack of systematic operational measurement tools for the conditions that facilitate DL development, especially contextualized scales that have undergone rigorous reliability and validity testing. As a result, although existing research can describe the developmental status of digital literacy, it remains limited in identifying specific problems and providing evidence for targeted interventions.

The formation of individual competence or literacy depends on the joint effects of multiple conditional factors, among which those that play a facilitative role essentially constitute supportive conditions for development. DL is inherently contextual and dynamic, while physical education is characterized by strong practical orientation and diverse technology application scenarios. Therefore, the development of DL among physical education majors depends more heavily on support from multiple conditions. It is thus necessary to move beyond a single-factor perspective and identify and structure relevant supportive elements from a holistic and systematic standpoint. Accordingly, this study defines these elements as Digital Literacy Education Support (DLES), referring to the system of supportive conditions and factors formed around the development of students’ DL.

In light of the above, this study is grounded in the dual identity characteristics and professional context of physical education majors. Based on a review of the literature and the collection of interview data, this study adopts grounded theory to code and analyze the interview transcripts, systematically extracting the key elements and relational structure of DLES for physical education majors. On this basis, a multi-level integrated educational support model is constructed, and a measurement scale for DLES is subsequently developed and validated. This study aims to provide theoretical evidence and a practical tool for promoting the digital literacy of physical education majors and to contribute to the advancement of educational digitalization strategies.

## 2 Construction of the digital literacy education support model for physical education majors

### 2.1 Construction method and procedure

To construct a DLES model for physical education majors in China, this study employed a qualitative research approach to obtain first-hand data. Semi-structured in-depth interviews were conducted to collect experiential data from participants in learning and practice contexts, thereby providing an empirical foundation for subsequent theoretical construction.

In terms of data analysis, grounded theory was used to systematically process the interview data. Grounded theory emphasizes the induction and generation of concepts and categories from empirical data (Charmaz and Thornberg, 2021). It is particularly suitable for research contexts in which the existing research foundation is relatively limited and the relevant elements of the research object have not yet been clearly defined. Therefore, grounded theory is highly consistent with the exploratory purpose of this study (Li and Ma, 2023).

During the implementation process, NVivo 20 (Lumivero) was used to code and manage the interview data, thereby improving the systematicity and traceability of data analysis. The specific procedure for model construction is presented in Figure 1.

**Figure 1:**
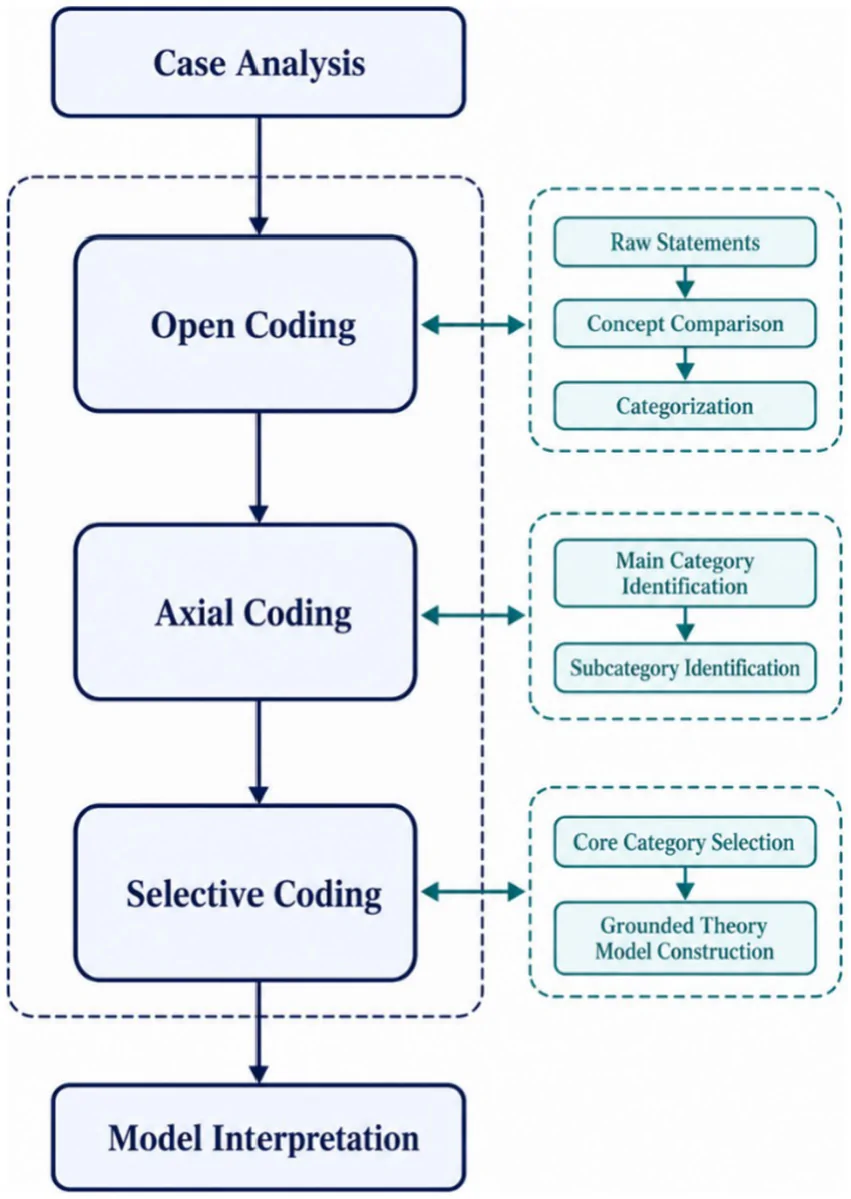
Flowchart of the model construction procedure.

### 2.2 Sample selection and data collection

Before the formal interviews, this study developed a preliminary interview guide based on the relevant literature and the research questions. The guide focused on DLES for physical education majors. Prior to the formal interviews, two experts in the field of physical education and three doctoral students were invited to participate in a small-scale discussion. They reviewed and refined the wording, logical structure, and contextual appropriateness of the interview questions to ensure that the guide was both sufficiently directive and easily understandable. The interview questions mainly included, but were not limited to, the following aspects: (1) How do you evaluate the current state of DL education for physical education majors at your institution? (2) What factors do you think mainly influence the DL of physical education majors? (3) Who do you think should be responsible for digital literacy education for physical education majors? (4) Do you think there is room for improvement in DL education for physical education majors? If so, in what specific aspects? (5) What are your suggestions for improving and optimizing DLES? During the interviews, semi-structured interviews were conducted based on the interview guide, and appropriate follow-up questions were asked according to participants’ responses to obtain richer and more in-depth data.

#### 2.2.1 Sample selection

In selecting the interview sample, this study followed the combined principles of purposive sampling and theoretical saturation sampling.

According to the requirements of purposive sampling, the inclusion criteria for participants were as follows: (1) having a certain understanding of the application of digital technologies in physical education practice; (2) being able to clearly understand the interview questions and express their views and ideas in a coherent manner; (3) being able to provide rich information relevant to the interview questions; and (4) having sufficient time and energy to participate in the interview and agreeing to audio recording throughout the process. On this basis, physical education majors were identified as the core interview group. The sample covered three types of institutions, namely normal universities, sport universities, and comprehensive universities, while also considering regional differences across eastern, central, western, and northeastern China to ensure sample diversity and representativeness. In addition, university teachers and counselors were included as supplementary interview participants to broaden the data sources from teaching and management perspectives. Following the principle of theoretical saturation sampling, interviews continued until no new concepts or categories emerged. At least three additional participants were then interviewed to further confirm saturation. Ultimately, 22 physical education majors and 12 university teachers and counselors were included as the formal interview sample. Detailed demographic characteristics of the participants are presented in Table 1.

**Table 1:** Characteristics of qualitative interview participants.

Participant group	Characteristics	Categories (*n*)
Physical education majors (*n* = 22)	Gender	Male (16), Female (6)
	Academic year	First year (5), Second year (5), Third year (7), Fourth year (5)
	University type	Sport universities (8), Normal universities (7), Comprehensive universities (7)
	Region	Eastern (7), Central (5), Western (6), Northeastern (4)
University teachers (*n* = 8)	Professional role	PE course instructors (8)
	University type	Sport universities (3), Normal universities (3), Comprehensive universities (2)
	Region	Eastern (3), Central (2), Western (2), Northeastern (1)
Counselors (*n* = 4)	Professional role	Student affairs counselors (4)
	University type	Sport universities (1), Normal universities (1), Comprehensive universities (2)
	Region	Eastern (1), Central (1), Western (1), Northeastern (1)

#### 2.2.2 Data collection

The collection of interview data lasted for 1 month. The specific process was as follows. First, during the formal interview stage, all participants were interviewed online. After obtaining informed consent from the participants, the interviews were audio-recorded throughout the process to ensure data completeness. Second, during the interviews, follow-up questions were asked when participants’ responses were unclear or ambiguous, so as to ensure the accuracy and completeness of the information collected. Third, after the interviews, the audio recordings were transcribed using iFLYTEK Hearing software version 9.4.4 (iFLYTEK Co., Ltd., China), followed by manual proofreading and revision to improve the accuracy and usability of the textual data. The finalized interview transcripts were then sent to the participants for confirmation to ensure that they truly and accurately reflected their views and ideas. Finally, the confirmed transcripts were imported into NVivo 20 (Lumivero) for systematic coding and categorization, with the aim of extracting the core elements of DLES for physical education majors.

### 2.3 Sample selection and data collection

#### 2.3.1 Open coding

In the open coding stage, this study conducted line-by-line coding and extracted a total of 833 original statements, namely original reference points. After labeling these statements and assigning *in vivo* concepts, 31 initial concepts were generated, including personal attitude, peer interaction behavior, hardware resources, social development needs, and conflicts between policy and practice, as shown in Table 2.

**Table 2:** Conceptual coding.

Original statement	Initial concept
It mainly depends on whether I personally want to engage with these things. It depends on my attitude. If I do not want to master or learn digital technologies, then no matter how good the external support is, it will be useless.	Personal attitude
I think the first factor depends on a person’s personality.	Personal personality
To put it plainly, no matter how good the external conditions are, if you do not have that drive or internal motivation, you will not be able to learn or use it well.	Intrinsic drive
For me, digital technology is not merely a tool. It is an added advantage that can help us differentiate ourselves from colleagues after entering the workforce.	Achievement orientation
I think that after learning this, it did not bring me much help in other aspects. Essentially, it only presents physical education practice in a digital way and allows us to learn by following it. However, it did not really improve my understanding of digital technology or help me acquire more digital skills.	Digital cognition
Sometimes when I really do not understand how to use certain software or platforms, I will ask classmates who know how to use them or seek help from teachers.	Help-seeking awareness
Sometimes teachers assign tasks that require digital tools, but I know many classmates simply use AI to generate the work or slightly revise others’ assignments before submitting them.	Integrity awareness
I think the most important purpose of using digital technology is to serve learning and future teaching effectiveness. We should not spread false information or violate others’ privacy.	Digital ethics awareness
It mainly depends on the teachers at the university. If teachers do not know much about this area, especially older teachers, they may be less likely to explain new digital society-related issues or tools.	Teachers’ digital literacy level
Whether we use certain digital tools largely depends on whether teachers use them. If teachers take the lead in using them and use them well, we will feel that these tools are useful and will be more willing to learn how to use them.	Teacher demonstration effect
Sometimes when we do not know how to use certain software, our first reaction is not to ask the teacher, but to see whether there are classmates around us who know how to use it. Having such peers around makes us more willing to try new digital tools.	Peers’ digital literacy level
Although teachers may explain some knowledge or operations in class, in actual practice we often rely on mutual help among classmates to understand them more quickly.	Peer interaction behavior
My parents have always urged me to learn more digital technologies. They say these are essential skills for future employment and teacher recruitment examinations. Their expectations in this regard have had a strong influence on me.	Parental expectations
Parents pay more attention to our sport skills. They may not pay much attention to digital skills, nor do they provide relevant platforms for us to learn them.	Parental recognition
Although some digital platforms or video analysis software are mentioned in our courses, the content is relatively general. It does not specifically address how to use them in physical education teaching, nor is it connected with our future teaching practice.	Curriculum content relevance
Sometimes the tasks assigned by teachers are suddenly too difficult. They only tell us what channels we can use to complete them and let us explore on our own, so we may just deal with the task superficially.	Curriculum task suitability
It depends on whether the university provides relevant digital education training for teachers.	Teacher education training
Although digital literacy is now emphasized, the university does not offer specialized training courses to teach us how to use digital technology for lesson preparation or teaching analysis. Most of the time, we explore by ourselves or even search for tutorials online.	Student education training
There are also facilities within the university. Some universities may not have enough equipment for students to use; for example, there may only be a multimedia player on the classroom blackboard.	Hardware resources
The software provided by the university is relatively limited, such as smart sport systems and digital resource management programs.	Software resources
Many things are now developing toward digitalization, such as smart campuses, online teaching, and data analysis software used in physical education classes. I think this is a major trend.	Digitalization trend
Since direct data output has become an important and advanced technical means, it is a technology that every educator must master; otherwise, one will fall behind the times.	Social development needs
Nowadays, many people use digital platforms to find learning resources, but some content is not professional and is not suitable for physical education majors. It may even lead us to learn incorrectly.	Quality of digital communication
In fact, there are many digital tools and platforms that can be used in physical education teaching and training, but we rarely hear anyone systematically introduce them, nor is it clearly explained how they can help us.	Digital education publicity
Whether there are relevant digital resources nearby matters. For example, our sport center is equipped with some basic digital facilities for public use.	Community digital resources
Although we can see that relevant digital resources exist, we need to go through a series of applications before we can actually use them.	Accessibility of digital resources
If national policies are issued, more people will pay attention to this issue, and relevant departments may then organize lectures or activities to help teachers and students improve their digital capabilities.	National policy guidance
Many classroom assignments require mobile phones to achieve good results, but we are required to hand in our phones before class.	Conflict between policy and practice
For physical education majors, we actually need practical training specifically focused on digital sport resources, but there is currently no systematic and well-developed training system.	Training system
Our digital works have no opportunities for display, and the university does not have an incentive mechanism. If there were bonus points or scholarships, we would definitely be more motivated.	Incentive system
Digital skills are rarely assessed in courses. Assessment mainly depends on assignments and examinations. If the use of digital tools were included in grades, students would pay more attention to it.	Evaluation and assessment system

The initial concepts were then clustered and categorized. Finally, 15 basic categories were formed, including psychological characteristics, peer support, digital resource support, and policy implementation. The basic categories, their corresponding initial concepts, category meanings, and naming rationale are presented in Table 3.

**Table 3:** Categorized coding.

Basic category	Initial concepts included	Meaning of the basic category
Basic category	Initial concepts; Initial included	Refers to students’ individual tendencies and psychological states in learning digital technologies, including their acceptance of new technologies, self-perception, and stable psychological characteristics.
Psychological characteristics	Personal attitude; personal personality	Refers to students’ subjective initiative, goal pursuit, and willingness to achieve self-improvement in the process of learning digital skills.
Learning ability	Digital cognition; help-seeking awareness	Refers to students’ ability to understand, master, and solve problems in digital environments, including tendencies toward active learning and help seeking.
Moral qualities	Integrity awareness; digital ethics awareness	Refers to the behavioral norms demonstrated by students when using digital technologies and resources, including honesty, respect for privacy, and compliance with ethical principles.
Teacher support	Teachers’ digital literacy level; teacher demonstration effect	Refers to whether teachers possess a certain level of DL and whether they provide demonstration and support for students.
Peer support	Peers’ digital literacy level; peer interaction behavior	Refers to the knowledge sharing, technical assistance, and demonstration influence that students obtain through peer interaction.
Family support	Parental expectations; parental recognition	Refers to parents’ understanding of and emphasis on digital education.
Curriculum support	Curriculum content relevance; curriculum task suitability	Refers to the extent to which course content matches the needs of physical education majors, as well as the practicality and difficulty suitability of digital tasks.
Training support	Teacher education training; student education training	Refers to whether universities provide systematic and targeted digital skills training for teachers and students.
Digital resource support	Hardware resources; software resources	Refers to the software and hardware infrastructure provided by universities for digital teaching, including digital devices, network conditions, teaching software, and platforms.
Social development trends	Digitalization trend; social development needs	Refers to students’ perceptions of the direction of educational digitalization, technological change, and the competencies required by future society.
Quality of the digital environment	Quality of digital communication; digital education publicity	Refers to the authenticity, practicality, dissemination effectiveness, and content appropriateness of digital education resources in the information environment.
Social resource support	Community digital resources; accessibility of digital resources	Refers to digital teaching equipment, platforms, activities, and their accessibility provided outside the university.
Policy implementation	National policy guidance; conflict between policy and practice	Refers to the degree of alignment between national top-level policy design for DL development and its actual implementation.
Rules and regulations	Training system; incentive system; evaluation and assessment system	Refers to the institutional arrangements established by national, local, and educational organizations to regulate and promote DL development.

#### 2.3.2 Axial coding

In the axial coding stage, this study further integrated the 15 basic categories and extracted five main categories: personal traits, symbiotic environment, school environment, social environment, and policy and institutional system, as shown in Table 4.

**Table 4:** Axial coding results.

Main category	Basic category	Meaning of the category relationship
Personal traits	Psychological characteristics	Personal traits constitute the internal foundation for physical education majors in the process of DL learning. They determine students’ cognitive attitudes toward digital technologies, learning willingness, and behavioral performance, and serve as the internal driving force for improving digital literacy. This category includes psychological characteristics, learning motivation, learning ability, and moral qualities.
	Learning motivation	
	Learning ability	
	Moral qualities	
Symbiotic environment	Teacher support	The symbiotic environment refers to the interactive support system formed between physical education majors and others during the learning process. These relational networks jointly shape students’ digital learning experiences and competence construction through guidance, collaboration, and encouragement. They are key external factors influencing the development of DL and include teacher support, peer support, and family support.
	Peer support	
	Family support	
School environment	Curriculum support	The school environment is the implementation carrier of digital literacy education. It determines whether physical education majors can systematically access, understand, and apply digital technologies. This category includes curriculum support, training support, and digital resource support.
	Training support	
	Digital resource support	
	Social development trends	The social environment is an important external support force for school education. It shapes the DL atmosphere and information ecology in which students are situated and serves as the broader background for extending the development of digital literacy. This category includes social development trends, quality of the digital environment, and social resource support.
	Quality of the digital environment	
	Social resource support	
Policy and institutional system	Policy implementation	The policy and institutional system refers to the guidance and regulation of digital literacy education within national and local educational governance systems. It determines whether DL education can be implemented and become effective in actual teaching practice, serving as an important bridge between top-level design and practical operation. This category includes policy implementation and rules and regulations.
	rules and regulations	

#### 2.3.3 Selective coding and model construction

Selective coding aims to identify the core category from a broad set of categories and systematically integrate diverse theoretical components through a coherent narrative storyline. Based on the previous two stages of coding, this study further explored and analyzed the complex relationships among the five main categories: personal traits, symbiotic environment, school environment, social environment, and policy and institutional system. The following storyline was then developed:

Under the guidance of national policies and institutional arrangements and driven by broader social environmental trends, DL education for physical education majors is influenced by both internal drivers and external environmental conditions. The policy and institutional system provides developmental direction and a regulatory foundation for schools and society, while the social environment offers resource provision and a climate of social recognition. Supported by policies and the social environment, the school environment establishes the basic field for DL education through curriculum design, training arrangements, and resource allocation. Within the symbiotic environment, teachers, peers, and families serve as important sources of educational support, influencing the digital learning behaviors and developmental pathways of physical education majors and forming a concrete system of social-interactive support. Personal traits, as the internal foundation, determine students’ motivation, learning ability, moral awareness, and other core dimensions related to digital technology use, and constitute the basis for whether DL can be internalized into a stable competence structure.

Based on this storyline, this study constructed a DLES model for physical education majors in China, as shown in Figure 2.

**Figure 2:**
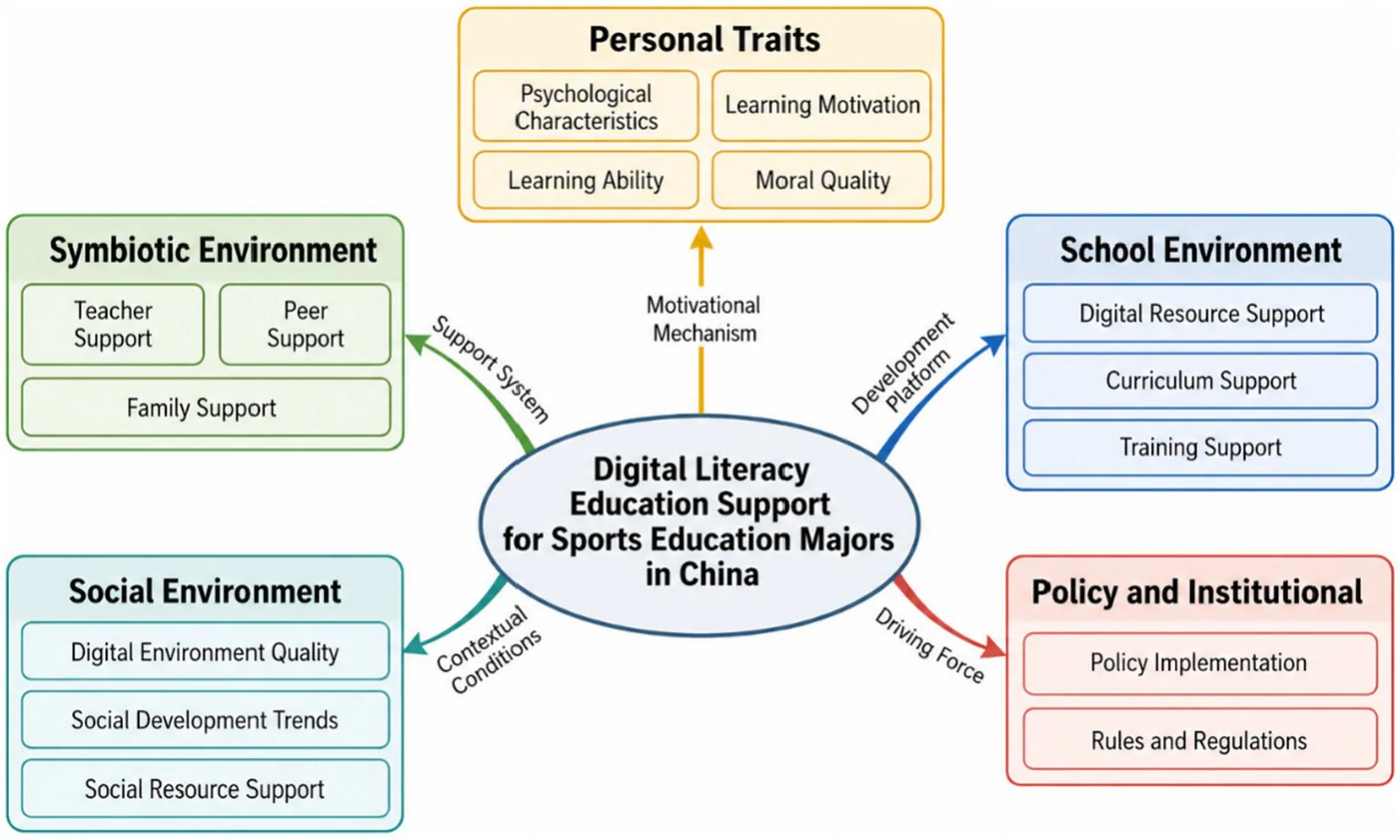
Digital literacy education support model for physical education majors in China.

#### 2.3.4 Selective coding and model construction

When the coding of data can no longer generate new categories or relationships, and more than three additional data sources are further examined without identifying any new categories or relationships, the theory constructed through coding can be considered to have reached saturation (Glaser and Strauss, 2017). In this study, theoretical saturation was used as one of the criteria for sample selection. Specifically, interviews were terminated when participants no longer provided new categories or relationships. Therefore, the final sample size indicates that theoretical saturation had been achieved.

#### 2.3.5 Coding reliability and trustworthiness analysis

To enhance the rigor of the grounded theory analysis and the credibility of the findings, multiple researchers, constant comparison, and researcher consensus were adopted as quality control strategies during the coding process. Three researchers participated in the data analysis process, including one researcher specializing in physical education, one researcher with experience in qualitative educational research, and one graduate student in physical education. All researchers possessed relevant knowledge of physical education, DL, or qualitative research methodology, enabling them to appropriately interpret the professional contexts reflected in the interview data. During open coding, two researchers independently coded the interview transcripts. Initial concepts were extracted through line-by-line analysis, and the identified concepts and categories were subsequently compared. Any discrepancies in coding results were discussed within the research team through re-examination of the original transcripts, reconsideration of conceptual meanings, and adjustment of category classifications until consensus was reached. During axial and selective coding, continuous comparative analysis was conducted to examine and refine the relationships among categories. The basic categories, main categories, and their relational structures were repeatedly reviewed and revised. Through collective discussion, five main categories—personal traits, symbiotic environment, school environment, social environment, and policy and institutional system—were finally identified, forming the theoretical model of DLES for physical education majors. In addition, NVivo 20 was used to document the coding process and analytical procedures, ensuring traceability. Investigator triangulation was employed to reduce potential individual interpretation bias and enhance the credibility of the grounded theory analysis (Zhang et al., 2021).

#### 2.3.6 Model interpretation

##### 2.3.6.1 Personal traits: the internal motivational mechanism for literacy formation

Personal traits constitute the internal psychological foundation for the formation of DL among physical education majors. They include four dimensions: psychological characteristics, learning motivation, learning ability, and moral qualities. These traits serve as the preconditions for physical education majors to engage actively in the learning and application of digital technologies. As a group with a dual identity, physical education majors’ personal traits not only influence their current learning performance but also determine whether they can meet the demands of future digitalized physical education practice. Psychological characteristics shape students’ acceptance of and stability in technology learning; learning motivation stimulates their active exploration of digital skills; learning ability supports their understanding and transfer of knowledge in complex environments; and moral qualities regulate their sense of responsibility and ethical judgment in technology use. Therefore, personal traits constitute the internal driving mechanism for the formation and development of DL among physical education majors and represent a key starting point for promoting identity transformation and literacy internalization.

##### 2.3.6.2 Symbiotic environment: the interpersonal support system for literacy construction

The symbiotic environment reflects the relational interactions between physical education majors and significant social others, including teachers, peers, and families. It consists of three dimensions: teacher support, peer support, and family support. This environment represents an indispensable social connection system in DL education for physical education majors. The symbiotic environment not only shapes students’ current learning behaviors but also provides an experiential foundation for constructing positive interaction and mutual development in their future identity as educators. Teacher support provides guidance and role modeling for the development of digital skills; peer support stimulates collaboration and constructive competition in technological exploration; and family support creates a digital learning atmosphere that extends beyond the classroom. These three types of support complement one another in their pathways of influence, strengthening students’ confidence in digital learning and willingness to engage in continuous learning. Thus, the symbiotic environment provides an important guarantee for the effective externalization of internal motivation.

##### 2.3.6.3 School environment: the institutional platform for literacy development

The school environment is the core field for DL education among physical education majors. It includes three dimensions: curriculum support, training support, and digital resource support. Together, these dimensions form an integrated supportive structure that provides a standardized and systematic practical carrier for DL education. On the one hand, physical education majors receive curriculum-based guidance as learners; on the other hand, they gradually simulate educational behaviors as future educators through practicum and field training. Curriculum support ensures students’ systematic understanding of digital education concepts and methods; training support strengthens the effective transfer of theoretical knowledge into teaching practice; and digital resource support provides authentic and diverse technological application scenarios. The coordinated integration of these three dimensions constitutes the institutional foundation and developmental pathway for DL construction. It promotes the spiral development of physical education majors from knowledge acquisition to competence output through multidimensional interaction.

##### 2.3.6.4 Social environment: the cultural context for literacy expansion

The social environment constitutes the broader cultural context and resource conditions for DL education among physical education majors. It includes three dimensions: social development trends, quality of the digital environment, and social resource support. These dimensions provide continuous driving forces for students’ learning behaviors and value cognition. The sociocultural system in which physical education majors are situated not only shapes their awareness of digital technology use but also defines the practical boundaries of literacy formation. Social development trends continuously reshape the forms and functional demands of educational technology, making digital literacy an increasingly core requirement for teacher professional development. The quality of the digital environment determines whether physical education majors can engage in autonomous exploration and technological expression within a safe, open, and shared information ecology. Social resource support injects vitality into the school education system through policy promotion, platform construction, and practical opportunities, thereby expanding individual developmental pathways. The evolution of the social environment not only provides a practical foundation for learning but also creates possible spaces for physical education majors to move from technology adapters to innovation leaders.

##### 2.3.6.5 Policy and institutional system: the institutional driving force for literacy formation

The policy and institutional system serves as the top-level design orientation for DL education among physical education majors. It includes two dimensions: policy implementation and rules and regulations. This system is the key link through which educational support mechanisms move from macro-level planning to micro-level practice. The dual identity of physical education majors requires them to continuously respond to the guiding demands of educational policies during their professional development. Policy implementation ensures the systematic embedding and practical realization of digital education concepts and provides institutional support for curriculum construction, competence assessment, and teaching standards. Rules and regulations guide physical education majors to develop technological literacy and teaching behaviors that meet the demands of the times through both normative and incentive mechanisms. Driven by national strategies such as educational digitalization and the “Double Reduction” policy, physical education majors face increasingly rigid requirements for digital competence. These requirements not only function as external driving forces but also stimulate the awakening of internal motivation and the willingness to construct digital literacy. As a bridge connecting the educational stage and the professional stage, the policy and institutional system provides an important institutional guarantee for physical education majors to achieve the identity transition from “tool users” to “instructional organizers.”

## 3 Development and validation of the digital literacy education support scale for physical education majors

Scale development and validation constitute a systematic and multi-stage process, the core purpose of which is to ensure that the measurement tool accurately reflects the meaning of the research construct and demonstrates sound psychometric properties (Hinkin, 1998). Specifically, a standardized scale development procedure typically includes the establishment of content validity, examination of scale structure, and validation of the scale within a theoretically relevant nomological network (Clark et al., 2020). In terms of methodological procedures, classic studies generally recommend following a systematic scale development paradigm, in which measurement items are generated based on the meaning of the construct and the reliability and validity of the scale are progressively tested through multi-stage data analyses (DeVellis and Thorpe, 2021; Nunnally, 1994).

Based on the above theoretical and methodological framework, and in combination with the DLES model for physical education majors constructed through grounded theory in the previous section, this study further conducted scale development and validation. Specifically, initial measurement items were first generated based on the qualitative findings, and expert review was then used to revise and optimize the item content to ensure adequate content validity. Second, using data from the pilot test and the formal survey, item analysis and exploratory factor analysis (EFA) were conducted to preliminarily examine the structure of the scale. Finally, confirmatory factor analysis (CFA), together with reliability and validity testing, was used to systematically evaluate the structural stability and measurement quality of the scale, thereby forming a measurement tool with sound reliability and validity.

### 3.1 Development of the initial item pool and content validity testing

In the process of scale development, questionnaire surveys are an important means of obtaining empirical data. Questionnaire design should closely align with the research questions and operationally measure the core variables. During item construction, existing mature scales should be consulted on the basis of a comprehensive review of the relevant literature, and localized revisions should be made according to the research context to ensure the appropriateness and validity of the measurement content. At the same time, item wording should be concise and clear, avoid ambiguity, and accurately reflect the meaning of the variables. Based on these principles, this study systematically designed and repeatedly revised the scale items to ensure both theoretical rigor and practical operability.

First, to ensure the scientific soundness and contextual suitability of the measurement tool, this study reviewed the research on DL and its influencing factors, with particular attention to studies consistent with the meaning of the variables examined in the present study. The content, dimensional structure, and application contexts of the scales used in these studies were analyzed in depth. On this basis, and in combination with the professional characteristics and dual-identity context of physical education majors, existing scales were appropriately revised and localized to ensure their validity and specificity in the physical education context. As a result, 32 initial items were generated for measuring DLES among physical education majors in China.

Second, to ensure the content validity of the scale, four domestic experts in sport science, including two experts in physical education and two experts in digital sport, were invited to review each item. The experts examined whether the item wording was concise and clear, whether ambiguity was avoided, and whether redundant expressions or statements inconsistent with the learning and practice contexts of physical education majors should be removed or revised. After this review, two items were deleted. To further verify the rationality of the remaining items, the level of item judgment agreement was calculated. The results showed that the judgment agreement for all remaining items exceeded 70%, indicating good agreement among expert evaluations. Finally, an initial measurement scale for DLES among physical education majors in China, consisting of 30 items, was developed, as shown in Table 5.

**Table 5:** Initial measurement scale for digital literacy education support among physical education majors in China.

Variable dimension	Item code	Measurement item	Reference source
Personal traits	A1	I am willing to explore how emerging digital technologies can be applied in physical education practice.	Venkatesh et al. (2012); Ryan and Deci (2000)
	A2	I can actively adapt to new challenges brought about by technological changes in physical education practice.	
	A3	I hope to improve my future competence in physical education practice by mastering digital technologies.	
	A4	I have a proactive willingness to use digital technologies as tools for instructional innovation.	
	A5	I can independently use digital tools to solve problems in physical education practice.	
	A6	I actively seek help from others to improve my ability to apply digital technologies.	
	A7	When using teaching platforms and digital resources, I pay attention to integrity and compliance with norms.	
	A8	I can consciously protect my own and others’ digital privacy and information security in physical education practice.	
Symbiotic environment	B1	Teachers often demonstrate the instructional application of digital tools in class.	Zhang (2021); Venkatesh et al. (2012)
	B2	I can learn the practical application of digital technologies from teachers’ teaching behaviors.	
	B3	I often improve my digital application ability through discussion and collaboration with classmates.	
	B4	Peers provide me with active support and operational demonstrations in the use of technologies.	
	B5	My family understands and supports my acquisition of career-related digital skills.	
	B6	My family members encourage me to participate in educational digitalization-related practical activities.	
School environment	C1	The course content is aligned with the digital literacy needs of future physical educators.	Man et al. (2024); Xing and Liu, (2023)
	C2	The tasks designed in courses can train my ability to solve technical problems in physical education practice.	
	C3	I have received specialized training on commonly used technological tools in physical education practice.	
	C4	My university provides specialized training related to digital literacy for physical education teachers.	
	C5	My university is equipped with digital hardware and a network environment suitable for physical education practice.	
	C6	I can access teaching resources such as sport-related digital software and analysis platforms.	
Social environment	D1	Social development trends make me aware of the importance of digital competence in physical education.	Martínez-Rico et al. (2022)
	D2	I understand the specific job-related requirements that society places on the digitalization of physical education.	
	D3	I often encounter high-quality publicity and reports on digital physical education.	
	D4	The dissemination of digital education content increases my motivation to learn digital technologies.	
	D5	I have participated in sport digital teaching activities or competitions organized outside the university.	
	D6	Outside the university, I can access rich sport-related digital equipment and application scenarios.	
Policy and institutional system	E1	During my learning process, I can clearly perceive the changes and support brought about by national digital education policies.	Ban (2024)
	E2	My university has specific measures and achievements in implementing national digital education policies.	
	E3	My university has incentive and assessment systems related to teachers’ digital teaching competence.	
	E4	My university has training arrangements for teacher education skills related to digital teaching.	

### 3.2 Testing and optimization of the initial scale

#### 3.2.1 Questionnaire design and data collection

Before the formal administration of the questionnaire, a small-scale pilot test was conducted to examine the reliability and validity of the questionnaire and to ensure the scientific soundness and applicability of the scale. The purpose of the pilot test was to preliminarily verify the rationality of the questionnaire structure, the clarity of item wording, and the stability of measurement through actual data collection, and to make necessary revisions and improvements based on the analytical results. The measurement scale adopted a five-point Likert scale, with responses ranging from 1 to 5: 1 = strongly disagree, 2 = disagree, 3 = uncertain, 4 = agree, and 5 = strongly agree. Higher scores indicated a higher level of agreement. The questionnaire was distributed via the Wenjuanxing platform to universities in eastern, central, western, and northeastern China that had been approved as national first-class undergraduate program construction sites for physical education. The participants were physical education majors from comprehensive universities, normal universities, and sport universities. During questionnaire administration, participants were informed of the research purpose, confidentiality principles, and instructions for completion. Data were collected online over a two-week period, resulting in 668 valid questionnaires.

#### 3.2.2 Item analysis

Reliability analysis and item discrimination analysis were used to examine the reliability and discriminative power of the scale items. The results showed that Cronbach’s *α* values for each dimension of the initial Digital Literacy Education Support Scale for physical education majors in China ranged from 0.712 to 0.894, all exceeding the threshold of 0.70, indicating acceptable reliability. Further analysis of the corrected item-total correlation (CITC) values showed that all items had CITC values greater than 0.50. In addition, according to the item discrimination analysis procedure, the total scores of the remaining 30 items were ranked, with the top 27% classified as the high-score group and the bottom 27% as the low-score group. Independent-samples t tests were then conducted to examine differences in mean scores for each item between the high- and low-score groups. The results showed significant differences between the two groups for all items (*p* < 0.001), indicating that the remaining 30 items of the initial DLES Scale for physical education majors in China had good discriminative power. Therefore, all items were retained.

#### 3.2.3 Exploratory factor analysis

Exploratory factor analysis was conducted on the remaining 30 items using SPSS 26.0. The Kaiser–Meyer–Olkin (KMO) test and Bartlett’s test of sphericity were first performed. The KMO value was 0.881 (>0.50), and Bartlett’s test of sphericity yielded an approximate chi-square value of 7648.344, which was significant (*p* < 0.001). These results indicated that the data were suitable for factor analysis and met the relevant criteria (Kaiser, 1974). On this basis, principal component analysis was used for factor extraction, and varimax orthogonal rotation was applied. EFA was conducted according to the criterion of retaining factors with eigenvalues greater than 1. During the analysis, no item showed a communality below 0.50, a factor loading below 0.50 on its corresponding common factor, or a cross-loading above 0.40. Therefore, no further items needed to be deleted.

Subsequently, principal component analysis with varimax rotation was used to extract factors with eigenvalues greater than 1. Based on the scree plot and the total variance explained, five factors were determined to be appropriate. The cumulative variance contribution rate was 58.119%, exceeding 50%, indicating that the extracted factors had good explanatory power for the variables. Convergence was achieved after four iterations of rotation, and the maximum factor loading of each item was greater than 0.50, meeting the required standard (Hinkin, 1998). The specific results are shown in Table 6.

**Table 6:** Exploratory factor analysis results of the digital literacy education support scale for physical education majors in china (pilot administration).

Questionnaire dimension	Item code	Component				
		1	2	3	4	5
Personal traits	A1	0.686				
	A2	0.741				
	A3	0.713				
	A4	0.704				
	A5	0.749				
	A6	0.677				
	A7	0.724				
	A8	0.671				
Symbiotic environment	B1		0.717			
	B2		0.659			
	B3		0.697			
	B4		0.751			
	B5		0.723			
	B6		0.683			
School environment	C1			0.799		
	C2			0.781		
	C3			0.770		
	C4			0.813		
	C5			0.797		
	C6			0.799		
Social environment	D1				0.696	
	D2				0.721	
	D3				0.688	
	D4				0.677	
	D5				0.636	
	D6				0.685	
Policy and institutional system	E1					0.798
	E2					0.782
	E3					0.775
	E4					0.752

*N* = 668. Extraction method: principal component analysis. Rotation method: varimax with Kaiser normalization.

### 3.3 Confirmatory factor analysis

AMOS 29.0 was used to conduct CFA to further examine the internal structural stability of the revised DLES Scale for physical education majors in China. The measurement scale developed after exploratory factor analysis, which contained 30 measurement items, was administered again through a new survey. A total of 1,498 electronic questionnaires were distributed via the Wenjuanxing platform. After invalid responses were excluded, 1,286 valid questionnaires were obtained, with an effective response rate of 85.85%.

To determine the optimal measurement model, CFA was used to compare the goodness-of-fit of different combinations of the five-factor model of DLES for physical education majors in China. The results showed that the five-factor model outperformed the other factor models across all fit indices. The model fit results indicated a good fit between the data and the hypothesized model: *χ*^2^/df = 2.725 (<3), RMSEA = 0.037 (<0.08), CFI = 0.968, TLI = 0.965, and IFI = 0.968, all exceeding 0.90. These indices met the recommended threshold criteria for structural equation modeling (Ryan and Deci, 2000), indicating that the five-factor model had good model fit. Therefore, the developed scale has a reasonable structure and can be used as the formal measurement scale for assessing DLES among physical education majors in China. Based on the above results, the five-factor model effectively reflects the conceptual characteristics of DLES for physical education majors in China. The formal measurement scale consists of five dimensions: personal traits, symbiotic environment, school environment, social environment, and policy and institutional system, with a total of 30 items. The specific results are shown in Table 7.

**Table 7:** Comparison of fit indices for the measurement models of digital literacy education support among physical education majors in China.

Model	*χ*^2^/df	RMSEA	IFI	TLI	CFI
One-factor model	22.058	0.128	0.601	0.571	0.601
Two-factor model a	18.975	0.118	0.660	0.634	0.660
Two-factor model b	16.344	0.109	0.710	0.687	0.710
Two-factor model c	16.552	0.110	0.706	0.683	0.706
Three-factor model	13.047	0.097	0.774	0.755	0.773
Four-factor model	7.546	0.071	0.878	0.867	0.878
Five-factor model	2.725	0.037	0.968	0.965	0.968
Reference criterion	<3	<0.08	>0.9	>0.9	>0.9

In the one-factor model, personal traits, symbiotic environment, school environment, social environment, and policy and institutional system were combined into a single factor. In two-factor model a, personal traits, symbiotic environment, school environment, and social environment were combined. In two-factor model b, personal traits, symbiotic environment, and school environment were combined into one factor, while social environment and policy and institutional system were combined into another factor. In two-factor model c, personal traits and symbiotic environment were combined into one factor, while school environment, social environment, and policy and institutional system were combined into another factor. In the three-factor model, personal traits, symbiotic environment, and school environment were combined. In the four-factor model, personal traits and symbiotic environment were combined.

### 3.4 Reliability and validity testing of the scale

The reliability of the scale was examined from three aspects: dimensional reliability, split-half reliability, and test–retest reliability. Dimensional reliability was assessed using Cronbach’s *α* coefficients. The results showed that the Cronbach’s *α* values of the dimensions ranged from 0.877 to 0.941, all exceeding 0.80. Split-half reliability was assessed using the odd–even split-half method, in which the items of each dimension were divided into two parts. The correlation coefficients between the scores of the two parts were calculated and then corrected using the Spearman–Brown formula to obtain the split-half reliability coefficients. The results showed that the split-half reliability coefficients ranged from 0.853 to 0.922. For test–retest reliability, 283 participants were retested after a four-week interval on a voluntary basis. The test–retest reliability coefficients between the two measurements ranged from 0.807 to 0.845, meeting the standard requirements and indicating good reliability (Nunnally, 1975). The reliability results are shown in Table 8.

**Table 8:** Reliability testing results of the scale.

Dimension	Cronbach’s *α*	Split-half reliability	Test–retest reliability
Personal traits	0.914	0.922	0.840
Symbiotic environment	0.885	0.892	0.807
School environment	0.893	0.901	0.815
Social environment	0.884	0.890	0.833
Policy and institutional system	0.854	0.853	0.845

Convergent validity was examined using composite reliability (CR) and average variance extracted (AVE). The results showed that the CR values of each dimension ranged from 0.901 to 0.930, all exceeding 0.80, while the AVE values ranged from 0.625 to 0.695, all exceeding 0.50. These results indicate that the scale demonstrated good convergent validity. Discriminant validity was assessed by comparing the square root of AVE with the inter-dimensional correlation coefficients. The results showed that the square root of AVE for each dimension was greater than the corresponding inter-dimensional correlation coefficients, meeting the recommended criteria. This indicates that the scale had good discriminant validity. The specific results are shown in Table 9.

**Table 9:** Results of convergent validity and discriminant validity.

Dimension	AVE	CR	1	2	3	4	5
1. Personal traits	0.625	0.930	(0.791)				
2. Symbiotic environment	0.636	0.913	0.543**	(0.797)			
3. School environment	0.652	0.918	0.480**	0.483**	(0.808)		
4. Social environment	0.634	0.912	0.418**	0.370**	0.406**	(0.796)	
5. Policy and institutional system	0.695	0.901	0.431**	0.358**	0.411**	0.474**	(0.834)

Values in parentheses on the diagonal are the square roots of AVE for each dimension. **p < 0.01.

## 4 Discussion

### 4.1 Systematic construction of the educational support model

The DLES model for physical education majors constructed in this study demonstrates strong rationality, mainly because it closely aligns with the characteristics of the research participants, the professional context, and the way in which the data were generated. First, the model fully considers the dual identity of physical education majors as both current learners and future educators. It understands DL within the continuous process of learning adaptation and future professional practice, thereby avoiding the limitation of explaining DL development solely from the perspective of general university students. Second, the model is closely connected to the practical characteristics of physical education, including its strong practice orientation and the embedding of digital technologies in teaching, training, and management scenarios. This makes the classification of support elements more professionally targeted. Third, this study used grounded theory to inductively extract categories from interview data, ensuring that the model was derived from authentic empirical materials rather than prior assumptions. This enhances the contextual explanatory power and empirical foundation of the model. Finally, the model integrates support elements across five levels: personal traits, symbiotic environment, school environment, social environment, and policy and institutional system. It therefore forms a hierarchical and holistic analytical framework that comprehensively presents the support logic underlying the development of DL among physical education majors.

Compared with existing studies, this study further advances the analytical perspective of DL research. Previous studies have mostly focused on digital literacy levels, technology integration competence, or single influencing factors, with analytical attention primarily placed on “what competencies students possess” or “whether a particular factor affects competence development” (Zhao et al., 2021). However, relatively limited attention has been paid to the overall support structure involved in the formation of DL. By contrast, this study shifts the analytical focus forward to supportive conditions and reveals that DL development is not driven by a single factor, but by the joint effects of multiple levels of support. In the specific context of physical education, students must adapt to digital learning while also preparing for future educational practice. The model constructed in this study better reflects the practical complexity of their DL development and helps explain how DL is generated and evolves within individual, relational, school, social, and institutional support conditions. Thus, this study not only enriches the object-specific perspective of DL research among physical education majors, but also promotes a shift in the field from “competence description” to “support structure explanation.”

### 4.2 Standardized development of the educational support scale

This study strictly followed the procedures of scale development and developed and validated the first standardized measurement scale for DLES among physical education majors. The scale consists of five dimensions and 30 items. The overall Cronbach’s *α* was 0.951. CFA showed that the five-factor model had good model fit (*χ*^2^/df = 2.896, CFI = 0.917, RMSEA = 0.075). The convergent validity of each dimension (AVE > 0.50) and discriminant validity, indicated by the square root of AVE being greater than the inter-dimensional correlation coefficients, both met recommended psychometric standards. The development of this scale transforms DLES from an abstract concept into a measurable, comparable, and diagnosable structure, thereby providing an instrumental basis for subsequent research and practical application.

Compared with existing measurement tools, the scale developed in this study shows clear differences in both measurement orientation and contextual adaptability. First, in terms of measurement orientation, existing studies have mainly focused on DL as an outcome variable. For example, relevant instruments have often been used to assess students’ DL levels (Tzafilkou et al., 2022; Wang et al., 2021), teachers’ technology integration competence, and teachers’ self-efficacy (Hatlevik et al., 2018; Chiu et al., 2024). Their core concern is “what abilities individuals possess.” In contrast, this study shifts the focus of measurement to “educational support” as an antecedent variable and operationally measures the supportive conditions that influence the formation of DL. Therefore, the scale can not only reflect individual developmental outcomes but also identify support structures and potential weaknesses in the cultivation process. Functionally, it represents a transition from an “outcome assessment tool” to a “process diagnostic tool.” Second, in terms of contextual adaptability, existing educational support measurement tools are mostly designed for general disciplinary contexts, and their items may not adequately capture the specific support needs of physical education majors (Park et al., 2025). In this study, the scale was generated and revised based on the real experiences of physical education majors, making the measurement content more closely aligned with specific contexts such as physical education teaching, training organization, and digital technology application. This not only improves the content validity of the scale but also enhances its explanatory power for the support structure of DL among physical education majors.

Furthermore, the educational support measured in this study does not represent general digital learning support for university students, but rather reflects the specific professional requirements of physical education training and practice. Due to the strong practical orientation of physical education, the development of DL among PE majors involves not only basic digital tool use but also the ability to apply digital technologies in professional contexts, including physical education teaching, sports training, and sport management. For example, with the increasing application of wearable fitness devices and intelligent physical fitness monitoring systems, future physical educators are expected to collect and interpret sport-related data, evaluate students’ physical activity performance, and adjust instructional strategies based on digital information. Similarly, sports motion analysis software, video-based feedback systems, and AI-assisted analysis tools provide new opportunities for identifying movement characteristics, delivering precise feedback, and facilitating individualized skill instruction. Therefore, dimensions such as digital resource support, curriculum support, and learning ability in the present model reflect not merely access to digital tools but also the capacity to integrate digital technologies into physical education practice. Moreover, the development of smart gym systems, digital physical education platforms, and intelligent sport management systems has expanded the professional application scenarios of DL among PE majors. Future physical educators need to engage not only in traditional teaching but also in sport data management, digital resource development, and intelligent learning environment construction. Accordingly, the present model emphasizes the importance of curriculum design, digital resource provision, teacher support, and institutional arrangements in cultivating PE majors’ DL. The developed scale therefore provides not only an assessment of students’ educational support environment but also a diagnostic tool for improving digital talent development systems in physical education programs.

### 4.3 Implications

From the perspective of an educational support system, this study constructed a multidimensional structural model of DLES for physical education majors and developed a corresponding standardized measurement tool. It therefore has substantive theoretical, methodological, and practical implications. Theoretically, this study extends the analysis of DL from an “individual competence-oriented” perspective to a “multilevel contextual support system” perspective. It goes beyond the dominant analytical paradigm in previous research, which has mainly relied on single-factor explanations, and deepens the understanding of the mechanism through which DL is generated. Methodologically, this study integrates grounded theory with quantitative validation, forming a complete research process from the identification of contextual elements to the testing of a structural model. This provides a transferable paradigm for combining exploratory and confirmatory approaches in educational research. Practically, by operationalizing educational support elements into a measurable structure, this study enables universities to systematically diagnose and dynamically evaluate the cultivation process of digital literacy, thereby promoting a shift in talent development from experience-driven to data-driven approaches.

### 4.4 Limitations and future directions

Although this study systematically explored model construction and scale development, several limitations remain. First, the sample was mainly drawn from Chinese universities, and the cross-cultural applicability of the findings requires further examination. Second, this study used cross-sectional data. Although the five-factor structure was verified, causal relationships could not be inferred, nor could the dynamic changes in support effects be tracked. Third, the scale data were mainly based on self-reports, which may involve a degree of subjective bias.

Future research can be extended in the following directions. First, cross-cultural comparative studies can be conducted to examine the generalizability and adaptability of the model. Second, longitudinal studies or experimental designs can be used to further explore the causal mechanisms and dynamic evolution through which educational support elements influence digital literacy development. Third, multi-source data, such as teacher evaluations, behavioral data, or learning process data, can be incorporated to improve the objectivity and explanatory power of measurement results.

## 5 Conclusion

Based on the dual identity of physical education majors as current learners and future educators, this study constructed a DLES model consisting of five dimensions: personal traits, symbiotic environment, school environment, social environment, and policy and institutional system. Through EFA and CFA, a 30-item measurement scale was developed. The scale met psychometric standards for reliability, convergent validity, and discriminant validity, and demonstrated a stable and reliable structure. The findings provide a theoretical framework and measurement tool for understanding the multilevel support mechanism underlying the formation of DL among physical education majors, and contribute to the advancement of educational digitalization strategies.

## Data Availability

The raw data supporting the conclusions of this article will be made available by the authors, without undue reservation.
